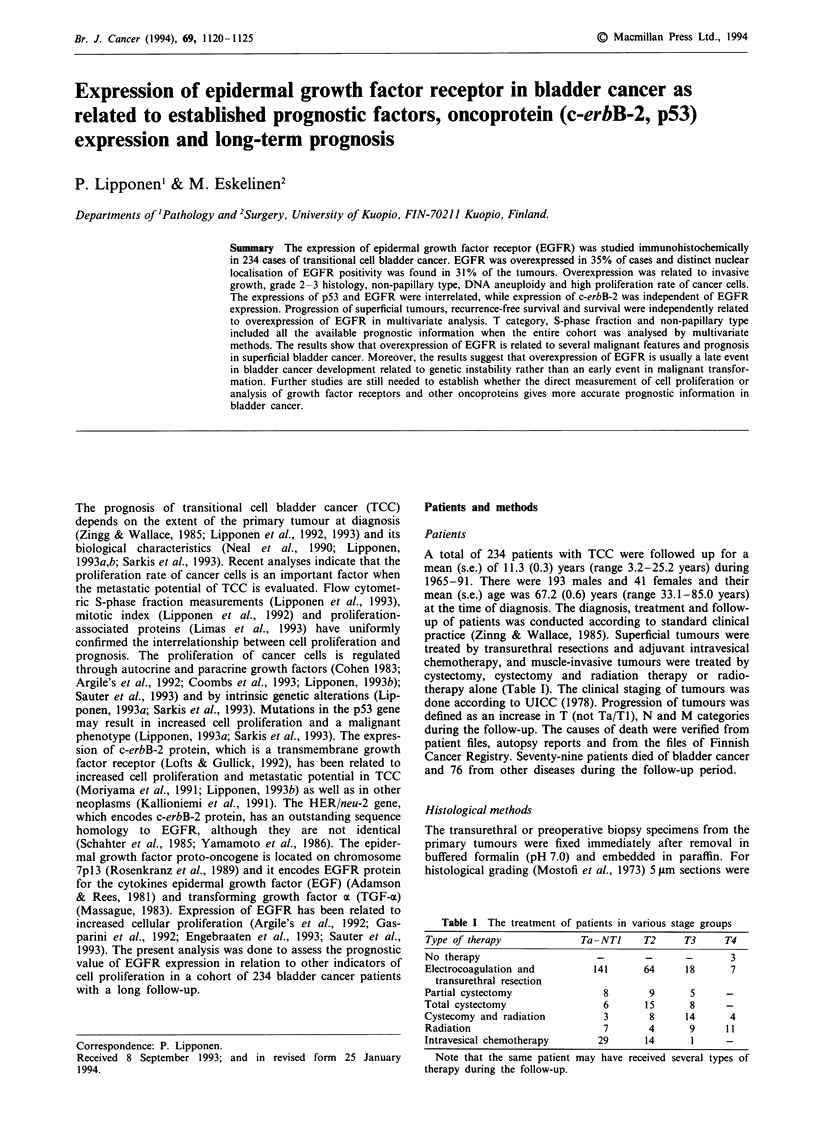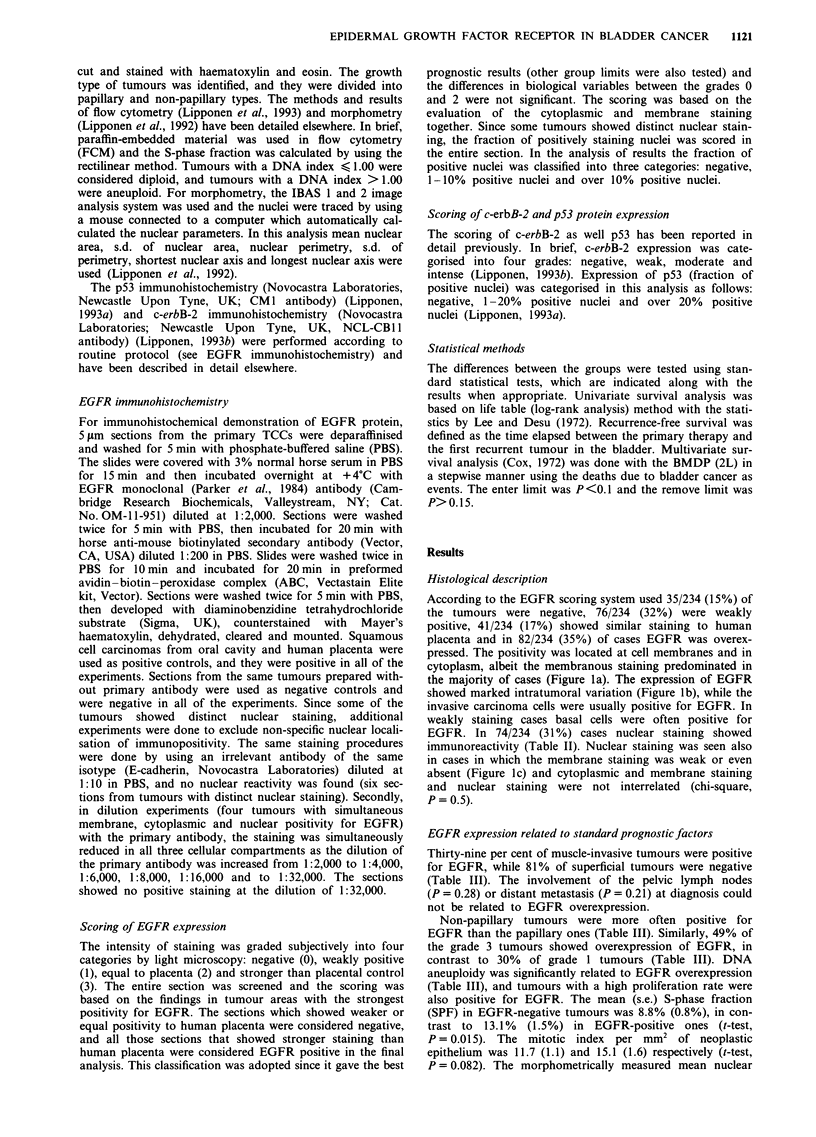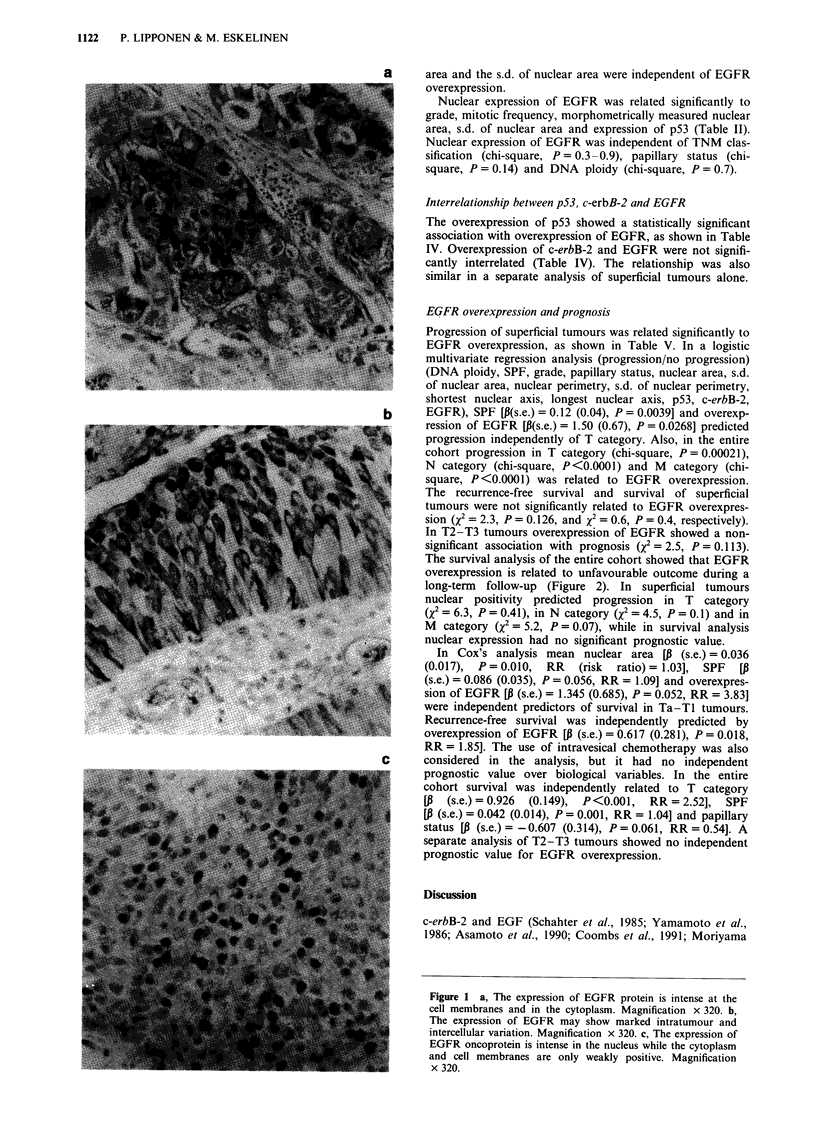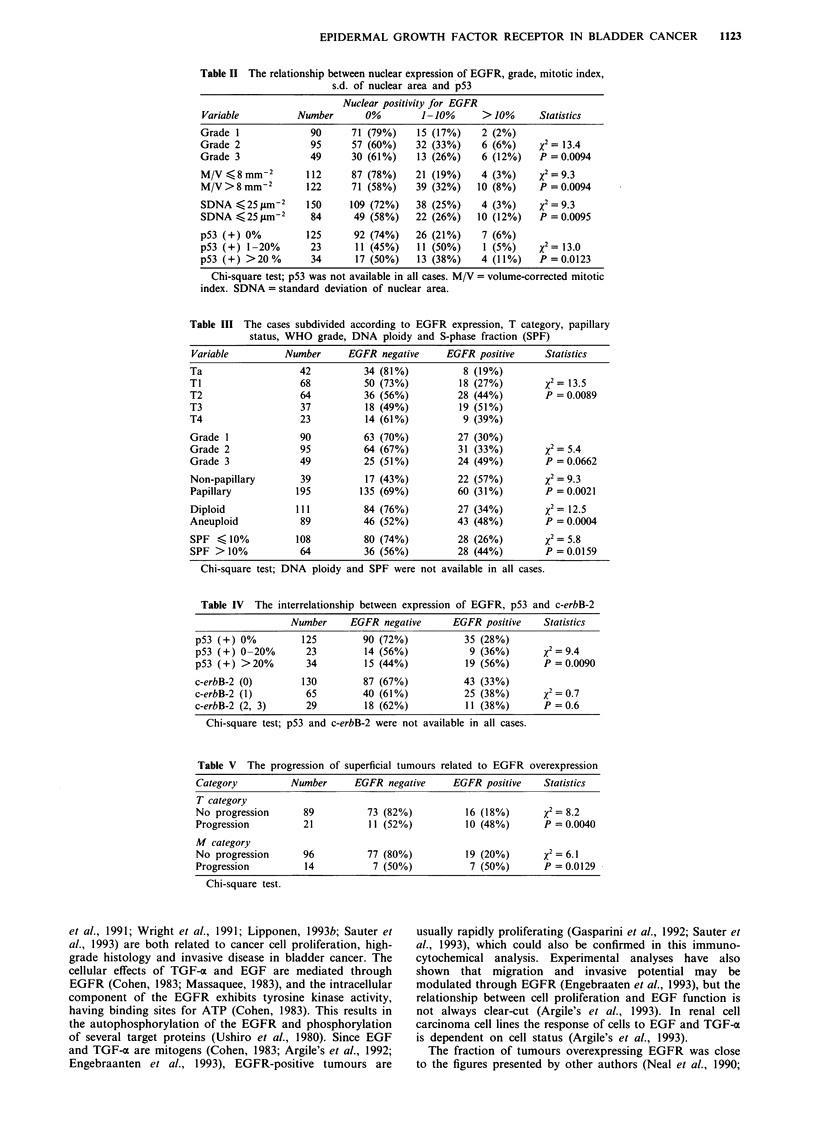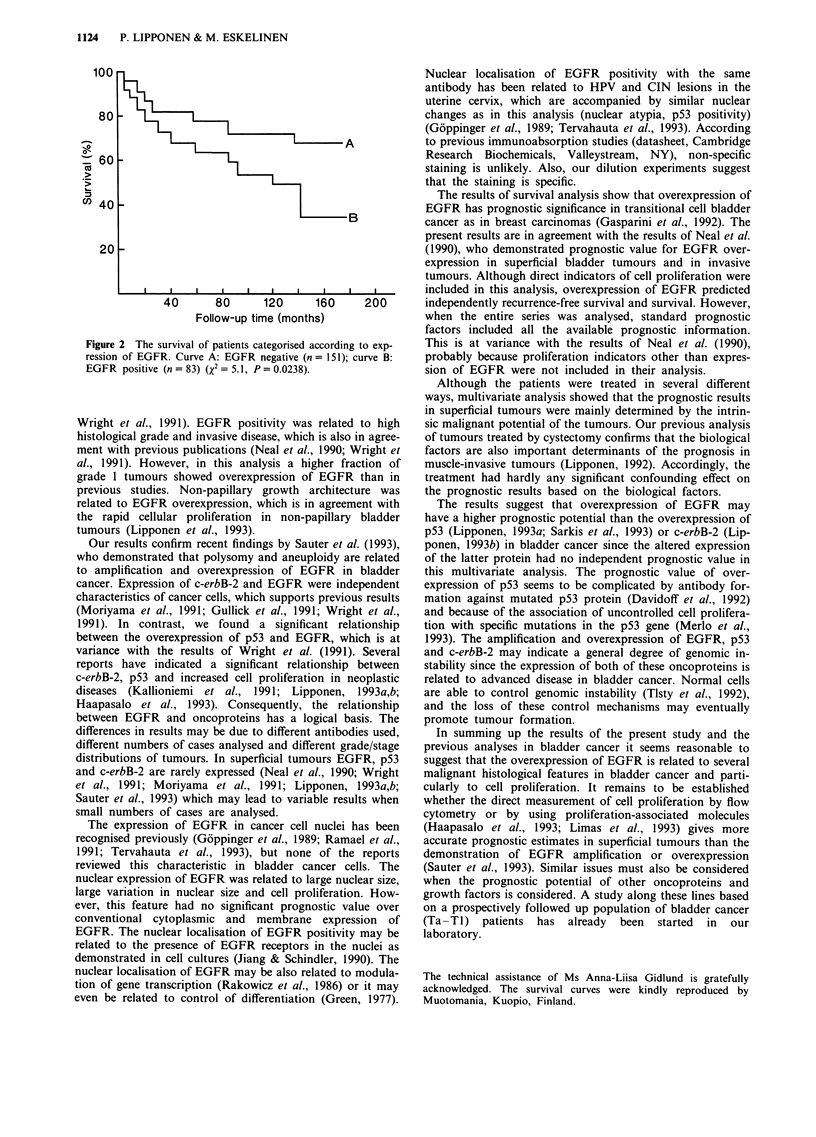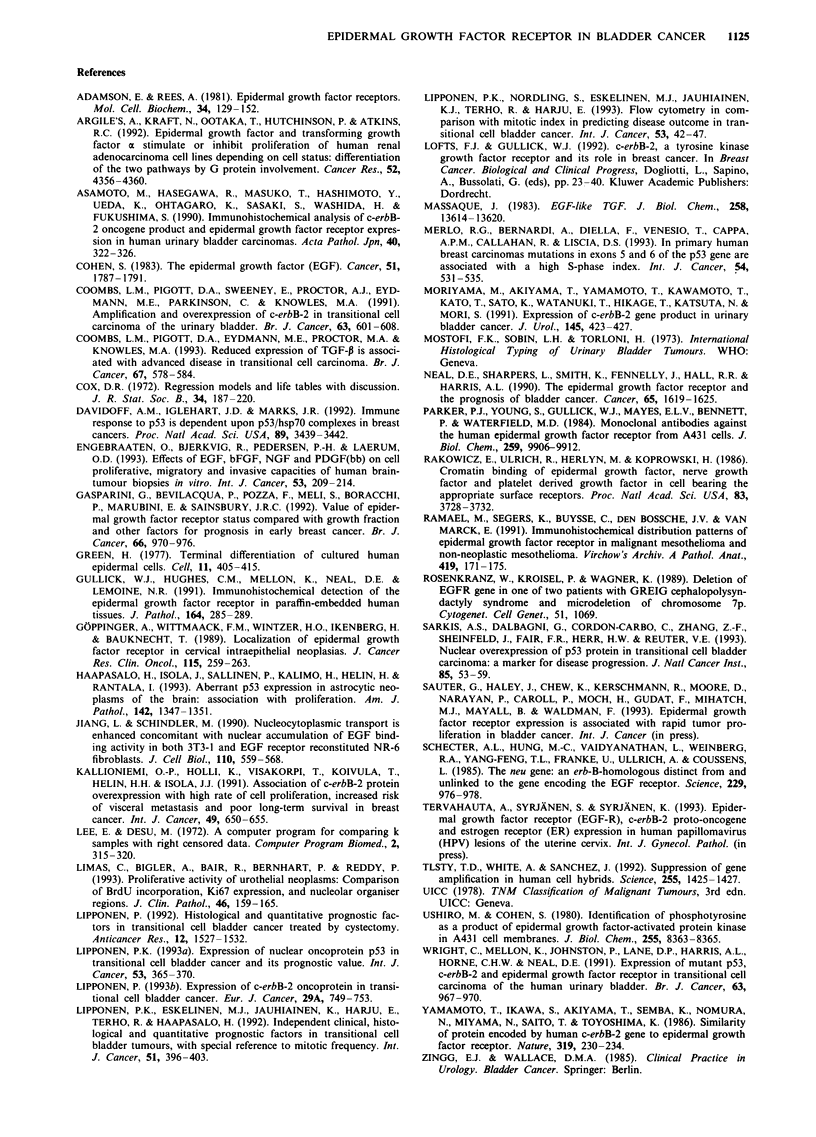# Expression of epidermal growth factor receptor in bladder cancer as related to established prognostic factors, oncoprotein (c-erbB-2, p53) expression and long-term prognosis.

**DOI:** 10.1038/bjc.1994.220

**Published:** 1994-06

**Authors:** P. Lipponen, M. Eskelinen

**Affiliations:** Department of Pathology, University of Kuopio, Finland.

## Abstract

**Images:**


*Br. J. Cancer* (1994), **69**, 1120–1125 © Macmillan Press Ltd., 1994

### Expression of epidermal growth factor receptor in bladder cancer as related to established prognostic factors, oncoprotein (c-*erb*B-2, p53) expression and long-term prognosis

P. Lipponen[1] & M. Eskelinen[2]

*Departments of [1]Pathology and [2]Surgery, University of Kuopio, FIN-70211 Kuopio, Finland.*

**Summary** The expression of epidermal growth factor receptor (EGFR) was studied immunohistochemically in 234 cases of transitional cell bladder cancer. EGFR was overexpressed in 35% of cases and distinct nuclear localisation of EGFR positivity was found in 31% of the tumours. Overexpression was related to invasive growth, grade 2–3 histology, non-papillary type, DNA aneuploidy and high proliferation rate of cancer cells. The expressions of p53 and EGFR were interrelated, while expression of c-*erb*B-2 was independent of EGFR expression. Progression of superficial tumours, recurrence-free survival and survival were independently related to overexpression of EGFR in multivariate analysis. T category, S-phase fraction and non-papillary type included all the available prognostic information when the entire cohort was analysed by multivariate methods. The results show that overexpression of EGFR is related to several malignant features and prognosis in superficial bladder cancer. Moreover, the results suggest that overexpression of EGFR is usually a late event in bladder cancer development related to genetic instability rather than an early event in malignant transformation. Further studies are still needed to establish whether the direct measurement of cell proliferation or analysis of growth factor receptors and other oncoproteins gives more accurate prognostic information in bladder cancer.

The prognosis of transitional cell bladder cancer (TCC) depends on the extent of the primary tumour at diagnosis (Zingg & Wallace, 1985; Lipponen *et al.*, 1992, 1993) and its biological characteristics (Neal *et al.*, 1990; Lipponen, 1993*a*,*b*; Sarkis *et al.*, 1993). Recent analyses indicate that the proliferation rate of cancer cells is an important factor when the metastatic potential of TCC is evaluated. Flow cytometric S-phase fraction measurements (Lipponen *et al.*, 1993), mitotic index (Lipponen *et al.*, 1992) and proliferation-associated proteins (Limas *et al.*, 1993) have uniformly confirmed the interrelationship between cell proliferation and prognosis. The proliferation of cancer cells is regulated through autocrine and paracrine growth factors (Cohen 1983; Argile's *et al.*, 1992; Coombs *et al.*, 1993; Lipponen, 1993*b*); Sauter *et al.*, 1993) and by intrinsic genetic alterations (Lipponen, 1993*a*; Sarkis *et al.*, 1993). Mutations in the p53 gene may result in increased cell proliferation and a malignant phenotype (Lipponen, 1993*a*; Sarkis *et al.*, 1993). The expression of c-*erb*B-2 protein, which is a transmembrane growth factor receptor (Lofts & Gullick, 1992), has been related to increased cell proliferation and metastatic potential in TCC (Moriyama *et al.*, 1991; Lipponen, 1993*b*) as well as in other neoplasms (Kallioniemi *et al.*, 1991). The HER/*neu*-2 gene, which encodes c-*erb*B-2 protein, has an outstanding sequence homology to EGFR, although they are not identical (Schahter *et al.*, 1985; Yamamoto *et al.*, 1986). The epidermal growth factor proto-oncogene is located on chromosome 7p13 (Rosenkranz *et al.*, 1989) and it encodes EGFR protein for the cytokines epidermal growth factor (EGF) (Adamson & Rees, 1981) and transforming growth factor α (TGF-α) (Massague, 1983). Expression of EGFR has been related to increased cellular proliferation (Argile's *et al.*, 1992; Gasparini *et al.*, 1992; Engebraaten *et al.*, 1993; Sauter *et al.*, 1993). The present analysis was done to assess the prognostic value of EGFR expression in relation to other indicators of cell proliferation in a cohort of 234 bladder cancer patients with a long follow-up.

Correspondence: P. Lipponen.
Received 8 September 1993; and in revised form 25 January 1994.

### Patients and methods

### *Patients*

A total of 234 patients with TCC were followed up for a mean (s.e.) of 11.3 (0.3) years (range 3.2–25.2 years) during 1965–91. There were 193 males and 41 females and their mean (s.e.) age was 67.2 (0.6) years (range 33.1–85.0 years) at the time of diagnosis. The diagnosis, treatment and follow-up of patients was conducted according to standard clinical practice (Zinng & Wallace, 1985). Superficial tumours were treated by transurethral resections and adjuvant intravesical chemotherapy, and muscle-invasive tumours were treated by cystectomy, cystectomy and radiation therapy or radiotherapy alone (Table I). The clinical staging of tumours was done according to UICC (1978). Progression of tumours was defined as an increase in T (not Ta/T1), N and M categories during the follow-up. The causes of death were verified from patient files, autopsy reports and from the files of Finnish Cancer Registry. Seventy-nine patients died of bladder cancer and 76 from other diseases during the follow-up period.

### *Histological methods*

The transurethral or preoperative biopsy specimens from the primary tumours were fixed immediately after removal in buffered formalin (pH 7.0) and embedded in paraffin. For histological grading (Mostofi *et al.*, 1973) 5 μm sections were

**Table I** The treatment of patients in various stage groups

| *Type of therapy* | *Ta–NT1* | *T2* | *T3* | *T4* |
|---|---|---|---|---|
| No therapy | – | – | – | 3 |
| Electrocoagulation and transurethral resection | 141 | 64 | 18 | 7 |
| Partial cystectomy | 8 | 9 | 5 | – |
| Total cystectomy | 6 | 15 | 8 | – |
| Cystecomy and radiation | 3 | 8 | 14 | 4 |
| Radiation | 7 | 4 | 9 | 11 |
| Intravesical chemotherapy | 29 | 14 | 1 | – |

Note that the same patient may have received several types of therapy during the follow-up.

cut and stained with haematoxylin and eosin. The growth type of tumours was identified, and they were divided into papillary and non-papillary types. The methods and results of flow cytometry (Lipponen *et al.*, 1993) and morphometry (Lipponen *et al.*, 1992) have been detailed elsewhere. In brief, paraffin-embedded material was used in flow cytometry (FCM) and the S-phase fraction was calculated by using the rectilinear method. Tumours with a DNA index $\leqslant 1.00$ were considered diploid, and tumours with a DNA index $> 1.00$ were aneuploid. For morphometry, the IBAS 1 and 2 image analysis system was used and the nuclei were traced by using a mouse connected to a computer which automatically calculated the nuclear parameters. In this analysis mean nuclear area, s.d. of nuclear area, nuclear perimetry, s.d. of perimetry, shortest nuclear axis and longest nuclear axis were used (Lipponen *et al.*, 1992).

The p53 immunohistochemistry (Novocastra Laboratories, Newcastle Upon Tyne, UK; CM1 antibody) (Lipponen, 1993*a*) and c-*erb*B-2 immunohistochemistry (Novocastra Laboratories; Newcastle Upon Tyne, UK, NCL-CB11 antibody) (Lipponen, 1993*b*) were performed according to routine protocol (see EGFR immunohistochemistry) and have been described in detail elsewhere.

*EGFR immunohistochemistry*

For immunohistochemical demonstration of EGFR protein, 5 μm sections from the primary TCCs were deparaffinised and washed for 5 min with phosphate-buffered saline (PBS). The slides were covered with 3% normal horse serum in PBS for 15 min and then incubated overnight at +4°C with EGFR monoclonal (Parker *et al.*, 1984) antibody (Cambridge Research Biochemicals, Valleystream, NY; Cat. No. OM-11-951) diluted at 1:2,000. Sections were washed twice for 5 min with PBS, then incubated for 20 min with horse anti-mouse biotinylated secondary antibody (Vector, CA, USA) diluted 1:200 in PBS. Slides were washed twice in PBS for 10 min and incubated for 20 min in preformed avidin–biotin–peroxidase complex (ABC, Vectastain Elite kit, Vector). Sections were washed twice for 5 min with PBS, then developed with diaminobenzidine tetrahydrochloride substrate (Sigma, UK), counterstained with Mayer's haematoxylin, dehydrated, cleared and mounted. Squamous cell carcinomas from oral cavity and human placenta were used as positive controls, and they were positive in all of the experiments. Sections from the same tumours prepared without primary antibody were used as negative controls and were negative in all of the experiments. Since some of the tumours showed distinct nuclear staining, additional experiments were done to exclude non-specific nuclear localisation of immunopositivity. The same staining procedures were done by using an irrelevant antibody of the same isotype (E-cadherin, Novocastra Laboratories) diluted at 1:10 in PBS, and no nuclear reactivity was found (six sections from tumours with distinct nuclear staining). Secondly, in dilution experiments (four tumours with simultaneous membrane, cytoplasmic and nuclear positivity for EGFR) with the primary antibody, the staining was simultaneously reduced in all three cellular compartments as the dilution of the primary antibody was increased from 1:2,000 to 1:4,000, 1:6,000, 1:8,000, 1:16,000 and to 1:32,000. The sections showed no positive staining at the dilution of 1:32,000.

*Scoring of EGFR expression*

The intensity of staining was graded subjectively into four categories by light microscopy: negative (0), weakly positive (1), equal to placenta (2) and stronger than placental control (3). The entire section was screened and the scoring was based on the findings in tumour areas with the strongest positivity for EGFR. The sections which showed weaker or equal positivity to human placenta were considered negative, and all those sections that showed stronger staining than human placenta were considered EGFR positive in the final analysis. This classification was adopted since it gave the best prognostic results (other group limits were also tested) and the differences in biological variables between the grades 0 and 2 were not significant. The scoring was based on the evaluation of the cytoplasmic and membrane staining together. Since some tumours showed distinct nuclear staining, the fraction of positively staining nuclei was scored in the entire section. In the analysis of results the fraction of positive nuclei was classified into three categories: negative, 1–10% positive nuclei and over 10% positive nuclei.

*Scoring of* c-erb*B-2 and p53 protein expression*

The scoring of c-*erb*B-2 as well p53 has been reported in detail previously. In brief, c-*erb*B-2 expression was categorised into four grades: negative, weak, moderate and intense (Lipponen, 1993*b*). Expression of p53 (fraction of positive nuclei) was categorised in this analysis as follows: negative, 1–20% positive nuclei and over 20% positive nuclei (Lipponen, 1993*a*).

*Statistical methods*

The differences between the groups were tested using standard statistical tests, which are indicated along with the results when appropriate. Univariate survival analysis was based on life table (log-rank analysis) method with the statistics by Lee and Desu (1972). Recurrence-free survival was defined as the time elapsed between the primary therapy and the first recurrent tumour in the bladder. Multivariate survival analysis (Cox, 1972) was done with the BMDP (2L) in a stepwise manner using the deaths due to bladder cancer as events. The enter limit was $P < 0.1$ and the remove limit was $P > 0.15$.

### Results

*Histological description*

According to the EGFR scoring system used 35/234 (15%) of the tumours were negative, 76/234 (32%) were weakly positive, 41/234 (17%) showed similar staining to human placenta and in 82/234 (35%) of cases EGFR was overexpressed. The positivity was located at cell membranes and in cytoplasm, albeit the membranous staining predominated in the majority of cases (Figure 1a). The expression of EGFR showed marked intratumoral variation (Figure 1b), while the invasive carcinoma cells were usually positive for EGFR. In weakly staining cases basal cells were often positive for EGFR. In 74/234 (31%) cases nuclear staining showed immunoreactivity (Table II). Nuclear staining was seen also in cases in which the membrane staining was weak or even absent (Figure 1c) and cytoplasmic and membrane staining and nuclear staining were not interrelated (chi-square, $P = 0.5$).

*EGFR expression related to standard prognostic factors*

Thirty-nine per cent of muscle-invasive tumours were positive for EGFR, while 81% of superficial tumours were negative (Table III). The involvement of the pelvic lymph nodes ($P = 0.28$) or distant metastasis ($P = 0.21$) at diagnosis could not be related to EGFR overexpression.

Non-papillary tumours were more often positive for EGFR than the papillary ones (Table III). Similarly, 49% of the grade 3 tumours showed overexpression of EGFR, in contrast to 30% of grade 1 tumours (Table III). DNA aneuploidy was significantly related to EGFR overexpression (Table III), and tumours with a high proliferation rate were also positive for EGFR. The mean (s.e.) S-phase fraction (SPF) in EGFR-negative tumours was 8.8% (0.8%), in contrast to 13.1% (1.5%) in EGFR-positive ones (*t*-test, $P = 0.015$). The mitotic index per $mm^2$ of neoplastic epithelium was 11.7 (1.1) and 15.1 (1.6) respectively (*t*-test, $P = 0.082$). The morphometrically measured mean nuclear area and the s.d. of nuclear area were independent of EGFR overexpression.

Nuclear expression of EGFR was related significantly to grade, mitotic frequency, morphometrically measured nuclear area, s.d. of nuclear area and expression of p53 (Table II). Nuclear expression of EGFR was independent of TNM classification (chi-square, $P = 0.3-0.9$), papillary status (chi-square, $P = 0.14$) and DNA ploidy (chi-square, $P = 0.7$).

*Interrelationship between p53, c-*erb*B-2 and EGFR*

The overexpression of p53 showed a statistically significant association with overexpression of EGFR, as shown in Table IV. Overexpression of c-*erb*B-2 and EGFR were not significantly interrelated (Table IV). The relationship was also similar in a separate analysis of superficial tumours alone.

*EGFR overexpression and prognosis*

Progression of superficial tumours was related significantly to EGFR overexpression, as shown in Table V. In a logistic multivariate regression analysis (progression/no progression) (DNA ploidy, SPF, grade, papillary status, nuclear area, s.d. of nuclear area, nuclear perimetry, s.d. of nuclear perimetry, shortest nuclear axis, longest nuclear axis, p53, c-*erb*B-2, EGFR), SPF [$\beta$(s.e.) = 0.12 (0.04), $P = 0.0039$] and overexpression of EGFR [$\beta$(s.e.) = 1.50 (0.67), $P = 0.0268$] predicted progression independently of T category. Also, in the entire cohort progression in T category (chi-square, $P = 0.00021$), N category (chi-square, $P < 0.0001$) and M category (chi-square, $P < 0.0001$) was related to EGFR overexpression. The recurrence-free survival and survival of superficial tumours were not significantly related to EGFR overexpression ($\chi^2 = 2.3$, $P = 0.126$, and $\chi^2 = 0.6$, $P = 0.4$, respectively). In T2–T3 tumours overexpression of EGFR showed a non-significant association with prognosis ($\chi^2 = 2.5$, $P = 0.113$). The survival analysis of the entire cohort showed that EGFR overexpression is related to unfavourable outcome during a long-term follow-up (Figure 2). In superficial tumours nuclear positivity predicted progression in T category ($\chi^2 = 6.3$, $P = 0.41$), in N category ($\chi^2 = 4.5$, $P = 0.1$) and in M category ($\chi^2 = 5.2$, $P = 0.07$), while in survival analysis nuclear expression had no significant prognostic value.

In Cox's analysis mean nuclear area [$\beta$ (s.e.) = 0.036 (0.017), $P = 0.010$, RR (risk ratio) = 1.03], SPF [$\beta$ (s.e.) = 0.086 (0.035), $P = 0.056$, RR = 1.09] and overexpression of EGFR [$\beta$ (s.e.) = 1.345 (0.685), $P = 0.052$, RR = 3.83] were independent predictors of survival in Ta–T1 tumours. Recurrence-free survival was independently predicted by overexpression of EGFR [$\beta$ (s.e.) = 0.617 (0.281), $P = 0.018$, RR = 1.85]. The use of intravesical chemotherapy was also considered in the analysis, but it had no independent prognostic value over biological variables. In the entire cohort survival was independently related to T category [$\beta$ (s.e.) = 0.926 (0.149), $P < 0.001$, RR = 2.52], SPF [$\beta$ (s.e.) = 0.042 (0.014), $P = 0.001$, RR = 1.04] and papillary status [$\beta$ (s.e.) = −0.607 (0.314), $P = 0.061$, RR = 0.54]. A separate analysis of T2–T3 tumours showed no independent prognostic value for EGFR overexpression.

### Discussion c-*erb*B-2 and EGF (Schahter *et al.*, 1985; Yamamoto *et al.*, 1986; Asamoto *et al.*, 1990; Coombs *et al.*, 1991; Moriyama

**Figure 1** **a**, The expression of EGFR protein is intense at the cell membranes and in the cytoplasm. Magnification × 320. **b**, The expression of EGFR may show marked intratumour and intercellular variation. Magnification × 320. **c**, The expression of EGFR oncoprotein is intense in the nucleus while the cytoplasm and cell membranes are only weakly positive. Magnification × 320.

**Table II** The relationship between nuclear expression of EGFR, grade, mitotic index, s.d. of nuclear area and p53

| *Variable* | *Number* | *Nuclear positivity for EGFR* 0% | 1–10% | >10% | *Statistics* |
|---|---|---|---|---|---|
| Grade 1 | 90 | 71 (79%) | 15 (17%) | 2 (2%) | |
| Grade 2 | 95 | 57 (60%) | 32 (33%) | 6 (6%) | $\chi^2 = 13.4$ |
| Grade 3 | 49 | 30 (61%) | 13 (26%) | 6 (12%) | $P = 0.0094$ |
| M/V $\leqslant 8\ mm^{-2}$ | 112 | 87 (78%) | 21 (19%) | 4 (3%) | $\chi^2 = 9.3$ |
| M/V $> 8\ mm^{-2}$ | 122 | 71 (58%) | 39 (32%) | 10 (8%) | $P = 0.0094$ |
| SDNA $\leqslant 25\ \mu m^{-2}$ | 150 | 109 (72%) | 38 (25%) | 4 (3%) | $\chi^2 = 9.3$ |
| SDNA $\leqslant 25\ \mu m^{-2}$ | 84 | 49 (58%) | 22 (26%) | 10 (12%) | $P = 0.0095$ |
| p53 (+) 0% | 125 | 92 (74%) | 26 (21%) | 7 (6%) | |
| p53 (+) 1–20% | 23 | 11 (45%) | 11 (50%) | 1 (5%) | $\chi^2 = 13.0$ |
| p53 (+) >20 % | 34 | 17 (50%) | 13 (38%) | 4 (11%) | $P = 0.0123$ |

Chi-square test; p53 was not available in all cases. M/V = volume-corrected mitotic index. SDNA = standard deviation of nuclear area.

**Table III** The cases subdivided according to EGFR expression, T category, papillary status, WHO grade, DNA ploidy and S-phase fraction (SPF)

| *Variable* | *Number* | *EGFR negative* | *EGFR positive* | *Statistics* |
|---|---|---|---|---|
| Ta | 42 | 34 (81%) | 8 (19%) | |
| T1 | 68 | 50 (73%) | 18 (27%) | $\chi^2 = 13.5$ |
| T2 | 64 | 36 (56%) | 28 (44%) | $P = 0.0089$ |
| T3 | 37 | 18 (49%) | 19 (51%) | |
| T4 | 23 | 14 (61%) | 9 (39%) | |
| Grade 1 | 90 | 63 (70%) | 27 (30%) | |
| Grade 2 | 95 | 64 (67%) | 31 (33%) | $\chi^2 = 5.4$ |
| Grade 3 | 49 | 25 (51%) | 24 (49%) | $P = 0.0662$ |
| Non-papillary | 39 | 17 (43%) | 22 (57%) | $\chi^2 = 9.3$ |
| Papillary | 195 | 135 (69%) | 60 (31%) | $P = 0.0021$ |
| Diploid | 111 | 84 (76%) | 27 (34%) | $\chi^2 = 12.5$ |
| Aneuploid | 89 | 46 (52%) | 43 (48%) | $P = 0.0004$ |
| SPF $\leqslant$10% | 108 | 80 (74%) | 28 (26%) | $\chi^2 = 5.8$ |
| SPF >10% | 64 | 36 (56%) | 28 (44%) | $P = 0.0159$ |

Chi-square test; DNA ploidy and SPF were not available in all cases.

**Table IV** The interrelationship between expression of EGFR, p53 and c-*erb*B-2

| | *Number* | *EGFR negative* | *EGFR positive* | *Statistics* |
|---|---|---|---|---|
| p53 (+) 0% | 125 | 90 (72%) | 35 (28%) | |
| p53 (+) 0–20% | 23 | 14 (56%) | 9 (36%) | $\chi^2 = 9.4$ |
| p53 (+) >20% | 34 | 15 (44%) | 19 (56%) | $P = 0.0090$ |
| c-*erb*B-2 (0) | 130 | 87 (67%) | 43 (33%) | |
| c-*erb*B-2 (1) | 65 | 40 (61%) | 25 (38%) | $\chi^2 = 0.7$ |
| c-*erb*B-2 (2, 3) | 29 | 18 (62%) | 11 (38%) | $P = 0.6$ |

Chi-square test; p53 and c-*erb*B-2 were not available in all cases.

**Table V** The progression of superficial tumours related to EGFR overexpression

| *Category* | *Number* | *EGFR negative* | *EGFR positive* | *Statistics* |
|---|---|---|---|---|
| *T category* | | | | |
| No progression | 89 | 73 (82%) | 16 (18%) | $\chi^2 = 8.2$ |
| Progression | 21 | 11 (52%) | 10 (48%) | $P = 0.0040$ |
| *M category* | | | | |
| No progression | 96 | 77 (80%) | 19 (20%) | $\chi^2 = 6.1$ |
| Progression | 14 | 7 (50%) | 7 (50%) | $P = 0.0129$ |

Chi-square test.

*et al.*, 1991; Wright *et al.*, 1991; Lipponen, 1993*b*; Sauter *et al.*, 1993) are both related to cancer cell proliferation, high-grade histology and invasive disease in bladder cancer. The cellular effects of TGF-α and EGF are mediated through EGFR (Cohen, 1983; Massaquee, 1983), and the intracellular component of the EGFR exhibits tyrosine kinase activity, having binding sites for ATP (Cohen, 1983). This results in the autophosphorylation of the EGFR and phosphorylation of several target proteins (Ushiro *et al.*, 1980). Since EGF and TGF-α are mitogens (Cohen, 1983; Argile's *et al.*, 1992; Engebraanten *et al.*, 1993), EGFR-positive tumours are usually rapidly proliferating (Gasparini *et al.*, 1992; Sauter *et al.*, 1993), which could also be confirmed in this immunocytochemical analysis. Experimental analyses have also shown that migration and invasive potential may be modulated through EGFR (Engebraaten *et al.*, 1993), but the relationship between cell proliferation and EGF function is not always clear-cut (Argile's *et al.*, 1993). In renal cell carcinoma cell lines the response of cells to EGF and TGF-α is dependent on cell status (Argile's *et al.*, 1993).

The fraction of tumours overexpressing EGFR was close to the figures presented by other authors (Neal *et al.*, 1990;

**Figure 2** The survival of patients categorised according to expression of EGFR. Curve A: EGFR negative ($n = 151$); curve B: EGFR positive ($n = 83$) ($\chi^2 = 5.1$, $P = 0.0238$).

Wright *et al.*, 1991). EGFR positivity was related to high histological grade and invasive disease, which is also in agreement with previous publications (Neal *et al.*, 1990; Wright *et al.*, 1991). However, in this analysis a higher fraction of grade 1 tumours showed overexpression of EGFR than in previous studies. Non-papillary growth architecture was related to EGFR overexpression, which is in agreement with the rapid cellular proliferation in non-papillary bladder tumours (Lipponen *et al.*, 1993).

Our results confirm recent findings by Sauter *et al.* (1993), who demonstrated that polysomy and aneuploidy are related to amplification and overexpression of EGFR in bladder cancer. Expression of c-*erb*B-2 and EGFR were independent characteristics of cancer cells, which supports previous results (Moriyama *et al.*, 1991; Gullick *et al.*, 1991; Wright *et al.*, 1991). In contrast, we found a significant relationship between the overexpression of p53 and EGFR, which is at variance with the results of Wright *et al.* (1991). Several reports have indicated a significant relationship between c-*erb*B-2, p53 and increased cell proliferation in neoplastic diseases (Kallioniemi *et al.*, 1991; Lipponen, 1993*a*,*b*; Haapasalo *et al.*, 1993). Consequently, the relationship between EGFR and oncoproteins has a logical basis. The differences in results may be due to different antibodies used, different numbers of cases analysed and different grade/stage distributions of tumours. In superficial tumours EGFR, p53 and c-*erb*B-2 are rarely expressed (Neal *et al.*, 1990; Wright *et al.*, 1991; Moriyama *et al.*, 1991; Lipponen, 1993*a*,*b*; Sauter *et al.*, 1993) which may lead to variable results when small numbers of cases are analysed.

The expression of EGFR in cancer cell nuclei has been recognised previously (Göppinger *et al.*, 1989; Ramael *et al.*, 1991; Tervahauta *et al.*, 1993), but none of the reports reviewed this characteristic in bladder cancer cells. The nuclear expression of EGFR was related to large nuclear size, large variation in nuclear size and cell proliferation. However, this feature had no significant prognostic value over conventional cytoplasmic and membrane expression of EGFR. The nuclear localisation of EGFR positivity may be related to the presence of EGFR receptors in the nuclei as demonstrated in cell cultures (Jiang & Schindler, 1990). The nuclear localisation of EGFR may be also related to modulation of gene transcription (Rakowicz *et al.*, 1986) or it may even be related to control of differentiation (Green, 1977). Nuclear localisation of EGFR positivity with the same antibody has been related to HPV and CIN lesions in the uterine cervix, which are accompanied by similar nuclear changes as in this analysis (nuclear atypia, p53 positivity) (Göppinger *et al.*, 1989; Tervahauta *et al.*, 1993). According to previous immunoabsorption studies (datasheet, Cambridge Research Biochemicals, Valleystream, NY), non-specific staining is unlikely. Also, our dilution experiments suggest that the staining is specific.

The results of survival analysis show that overexpression of EGFR has prognostic significance in transitional cell bladder cancer as in breast carcinomas (Gasparini *et al.*, 1992). The present results are in agreement with the results of Neal *et al.* (1990), who demonstrated prognostic value for EGFR overexpression in superficial bladder tumours and in invasive tumours. Although direct indicators of cell proliferation were included in this analysis, overexpression of EGFR predicted independently recurrence-free survival and survival. However, when the entire series was analysed, standard prognostic factors included all the available prognostic information. This is at variance with the results of Neal *et al.* (1990), probably because proliferation indicators other than expression of EGFR were not included in their analysis.

Although the patients were treated in several different ways, multivariate analysis showed that the prognostic results in superficial tumours were mainly determined by the intrinsic malignant potential of the tumours. Our previous analysis of tumours treated by cystectomy confirms that the biological factors are also important determinants of the prognosis in muscle-invasive tumours (Lipponen, 1992). Accordingly, the treatment had hardly any significant confounding effect on the prognostic results based on the biological factors.

The results suggest that overexpression of EGFR may have a higher prognostic potential than the overexpression of p53 (Lipponen, 1993*a*; Sarkis *et al.*, 1993) or c-*erb*B-2 (Lipponen, 1993*b*) in bladder cancer since the altered expression of the latter protein had no independent prognostic value in this multivariate analysis. The prognostic value of overexpression of p53 seems to be complicated by antibody formation against mutated p53 protein (Davidoff *et al.*, 1992) and because of the association of uncontrolled cell proliferation with specific mutations in the p53 gene (Merlo *et al.*, 1993). The amplification and overexpression of EGFR, p53 and c-*erb*B-2 may indicate a general degree of genomic instability since the expression of both of these oncoproteins is related to advanced disease in bladder cancer. Normal cells are able to control genomic instability (Tlsty *et al.*, 1992), and the loss of these control mechanisms may eventually promote tumour formation.

In summing up the results of the present study and the previous analyses in bladder cancer it seems reasonable to suggest that the overexpression of EGFR is related to several malignant histological features in bladder cancer and particularly to cell proliferation. It remains to be established whether the direct measurement of cell proliferation by flow cytometry or by using proliferation-associated molecules (Haapasalo *et al.*, 1993; Limas *et al.*, 1993) gives more accurate prognostic estimates in superficial tumours than the demonstration of EGFR amplification or overexpression (Sauter *et al.*, 1993). Similar issues must also be considered when the prognostic potential of other oncoproteins and growth factors is considered. A study along these lines based on a prospectively followed up population of bladder cancer (Ta–T1) patients has already been started in our laboratory.

The technical assistance of Ms Anna-Liisa Gidlund is gratefully acknowledged. The survival curves were kindly reproduced by Muotomania, Kuopio, Finland.

### References

ADAMSON, E. & REES, A. (1981). Epidermal growth factor receptors. *Mol. Cell. Biochem.*, **34**, 129–152.

ARGILE'S, A., KRAFT, N., OOTAKA, T., HUTCHINSON, P. & ATKINS, R.C. (1992). Epidermal growth factor and transforming growth factor α stimulate or inhibit proliferation of human renal adenocarcinoma cell lines depending on cell status: differentiation of the two pathways by G protein involvement. *Cancer Res.*, **52**, 4356–4360.

ASAMOTO, M., HASEGAWA, R., MASUKO, T., HASHIMOTO, Y., UEDA, K., OHTAGARO, K., SASAKI, S., WASHIDA, H. & FUKUSHIMA, S. (1990). Immunohistochemical analysis of c-*erb*B-2 oncogene product and epidermal growth factor receptor expression in human urinary bladder carcinomas. *Acta Pathol. Jpn*, **40**, 322–326.

COHEN, S. (1983). The epidermal growth factor (EGF). *Cancer*, **51**, 1787–1791.

COOMBS, L.M., PIGOTT, D.A., SWEENEY, E., PROCTOR, A.J., EYDMANN, M.E., PARKINSON, C. & KNOWLES, M.A. (1991). Amplification and overexpression of c-*erb*B-2 in transitional cell carcinoma of the urinary bladder. *Br. J. Cancer*, **63**, 601–608.

COOMBS, L.M., PIGOTT, D.A., EYDMANN, M.E., PROCTOR, M.A. & KNOWLES, M.A. (1993). Reduced expression of TGF-β is associated with advanced disease in transitional cell carcinoma. *Br. J. Cancer*, **67**, 578–584.

COX, D.R. (1972). Regression models and life tables with discussion. *J. R. Stat. Soc. B.*, **34**, 187–220.

DAVIDOFF, A.M., IGLEHART, J.D. & MARKS, J.R. (1992). Immune response to p53 is dependent upon p53/hsp70 complexes in breast cancers. *Proc. Natl Acad. Sci. USA*, **89**, 3439–3442.

ENGEBRAATEN, O., BJERKVIG, R., PEDERSEN, P.-H. & LAERUM, O.D. (1993). Effects of EGF, bFGF, NGF and PDGF(bb) on cell proliferative, migratory and invasive capacities of human braintumour biopsies *in vitro*. *Int. J. Cancer*, **53**, 209–214.

GASPARINI, G., BEVILACQUA, P., POZZA, F., MELI, S., BORACCHI, P., MARUBINI, E. & SAINSBURY, J.R.C. (1992). Value of epidermal growth factor receptor status compared with growth fraction and other factors for prognosis in early breast cancer. *Br. J. Cancer*, **66**, 970–976.

GREEN, H. (1977). Terminal differentiation of cultured human epidermal cells. *Cell*, **11**, 405–415.

GULLICK, W.J., HUGHES, C.M., MELLON, K., NEAL, D.E. & LEMOINE, N.R. (1991). Immunohistochemical detection of the epidermal growth factor receptor in paraffin-embedded human tissues. *J. Pathol.*, **164**, 285–289.

GÖPPINGER, A., WITTMAACK, F.M., WINTZER, H.O., IKENBERG, H. & BAUKNECHT, T. (1989). Localization of epidermal growth factor receptor in cervical intraepithelial neoplasias. *J. Cancer Res. Clin. Oncol.*, **115**, 259–263.

HAAPASALO, H., ISOLA, J., SALLINEN, P., KALIMO, H., HELIN, H. & RANTALA, I. (1993). Aberrant p53 expression in astrocytic neoplasms of the brain: association with proliferation. *Am. J. Pathol.*, **142**, 1347–1351.

JIANG, L. & SCHINDLER, M. (1990). Nucleocytoplasmic transport is enhanced concomitant with nuclear accumulation of EGF binding activity in both 3T3-1 and EGF receptor reconstituted NR-6 fibroblasts. *J. Cell Biol.*, **110**, 559–568.

KALLIONIEMI, O.-P., HOLLI, K., VISAKORPI, T., KOIVULA, T., HELIN, H.H. & ISOLA, J.J. (1991). Association of c-*erb*B-2 protein overexpression with high rate of cell proliferation, increased risk of visceral metastasis and poor long-term survival in breast cancer. *Int. J. Cancer*, **49**, 650–655.

LEE, E. & DESU, M. (1972). A computer program for comparing k samples with right censored data. *Computer Program Biomed.*, **2**, 315–320.

LIMAS, C., BIGLER, A., BAIR, R., BERNHART, P. & REDDY, P. (1993). Proliferative activity of urothelial neoplasms: Comparison of BrdU incorporation, Ki67 expression, and nucleolar organiser regions. *J. Clin. Pathol.*, **46**, 159–165.

LIPPONEN, P. (1992). Histological and quantitative prognostic factors in transitional cell bladder cancer treated by cystectomy. *Anticancer Res.*, **12**, 1527–1532.

LIPPONEN, P.K. (1993*a*). Expression of nuclear oncoprotein p53 in transitional cell bladder cancer and its prognostic value. *Int. J. Cancer*, **53**, 365–370.

LIPPONEN, P. (1993*b*). Expression of c-*erb*B-2 oncoprotein in transitional cell bladder cancer. *Eur. J. Cancer*, **29A**, 749–753.

LIPPONEN, P.K., ESKELINEN, M.J., JAUHIAINEN, K., HARJU, E., TERHO, R. & HAAPASALO, H. (1992). Independent clinical, histological and quantitative prognostic factors in transitional cell bladder tumours, with special reference to mitotic frequency. *Int. J. Cancer*, **51**, 396–403.

LIPPONEN, P.K., NORDLING, S., ESKELINEN, M.J., JAUHIAINEN, K.J., TERHO, R. & HARJU, E. (1993). Flow cytometry in comparison with mitotic index in predicting disease outcome in transitional cell bladder cancer. *Int. J. Cancer*, **53**, 42–47.

LOFTS, F.J. & GULLICK, W.J. (1992). c-*erb*B-2, a tyrosine kinase growth factor receptor and its role in breast cancer. In *Breast Cancer. Biological and Clinical Progress*, Dogliotti, L., Sapino, A., Bussolati, G. (eds), pp. 23–40. Kluwer Academic Publishers: Dordrecht.

MASSAQUE, J. (1983). *EGF-like TGF. J. Biol. Chem.*, **258**, 13614–13620.

MERLO, R.G., BERNARDI, A., DIELLA, F., VENESIO, T., CAPPA, A.P.M., CALLAHAN, R. & LISCIA, D.S. (1993). In primary human breast carcinomas mutations in exons 5 and 6 of the p53 gene are associated with a high S-phase index. *Int. J. Cancer*, **54**, 531–535.

MORIYAMA, M., AKIYAMA, T., YAMAMOTO, T., KAWAMOTO, T., KATO, T., SATO, K., WATANUKI, T., HIKAGE, T., KATSUTA, N. & MORI, S. (1991). Expression of c-*erb*B-2 gene product in urinary bladder cancer. *J. Urol.*, **145**, 423–427.

MOSTOFI, F.K., SOBIN, L.H. & TORLONI, H. (1973). *International Histological Typing of Urinary Bladder Tumours*. WHO: Geneva.

NEAL, D.E., SHARPERS, L., SMITH, K., FENNELLY, J., HALL, R.R. & HARRIS, A.L. (1990). The epidermal growth factor receptor and the prognosis of bladder cancer. *Cancer*, **65**, 1619–1625.

PARKER, P.J., YOUNG, S., GULLICK, W.J., MAYES, E.L.V., BENNETT, P. & WATERFIELD, M.D. (1984). Monoclonal antibodies against the human epidermal growth factor receptor from A431 cells. *J. Biol. Chem.*, **259**, 9906–9912.

RAKOWICZ, E., ULRICH, R., HERLYN, M. & KOPROWSKI, H. (1986). Cromatin binding of epidermal growth factor, nerve growth factor and platelet derived growth factor in cell bearing the appropriate surface receptors. *Proc. Natl Acad. Sci. USA*, **83**, 3728–3732.

RAMAEL, M., SEGERS, K., BUYSSE, C., DEN BOSSCHE, J.V. & VAN MARCK, E. (1991). Immunohistochemical distribution patterns of epidermal growth factor receptor in malignant mesothelioma and non-neoplastic mesothelioma. *Virchow's Archiv. A Pathol. Anat.*, **419**, 171–175.

ROSENKRANZ, W., KROISEL, P. & WAGNER, K. (1989). Deletion of EGFR gene in one of two patients with GREIG cephalopolysyndactyly syndrome and microdeletion of chromosome 7p. *Cytogenet. Cell Genet.*, 51, 1069.

SARKIS, A.S., DALBAGNI, G., CORDON-CARBO, C., ZHANG, Z.-F., SHEINFELD, J., FAIR, F.R., HERR, H.W. & REUTER, V.E. (1993). Nuclear overexpression of p53 protein in transitional cell bladder carcinoma: a marker for disease progression. *J. Natl Cancer Inst.*, **85**, 53–59.

SAUTER, G., HALEY, J., CHEW, K., KERSCHMANN, R., MOORE, D., NARAYAN, P., CAROLL, P., MOCH, H., GUDAT, F., MIHATCH, M.J., MAYALL, B. & WALDMAN, F. (1993). Epidermal growth factor receptor expression is associated with rapid tumor proliferation in bladder cancer. *Int. J. Cancer* (in press).

SCHECTER, A.L., HUNG, M.-C., VAIDYANATHAN, L., WEINBERG, R.A., YANG-FENG, T.L., FRANKE, U., ULLRICH, A. & COUSSENS, L. (1985). The *neu* gene: an *erb*-B-homologous distinct from and unlinked to the gene encoding the EGF receptor. *Science*, **229**, 976–978.

TERVAHAUTA, A., SYRJÄNEN, S. & SYRJÄNEN, K. (1993). Epidermal growth factor receptor (EGF-R), c-*erb*B-2 proto-oncogene and estrogen receptor (ER) expression in human papillomavirus (HPV) lesions of the uterine cervix. *Int. J. Gynecol. Pathol.* (in press).

TLSTY, T.D., WHITE, A. & SANCHEZ, J. (1992). Suppression of gene amplification in human cell hybrids. *Science*, **255**, 1425–1427.

UICC (1978). *TNM Classification of Malignant Tumours*, 3rd edn. UICC: Geneva.

USHIRO, M. & COHEN, S. (1980). Identification of phosphotyrosine as a product of epidermal growth factor-activated protein kinase in A431 cell membranes. *J. Biol. Chem.*, **255**, 8363–8365.

WRIGHT, C., MELLON, K., JOHNSTON, P., LANE, D.P., HARRIS, A.L., HORNE, C.H.W. & NEAL, D.E. (1991). Expression of mutant p53, c-*erb*B-2 and epidermal growth factor receptor in transitional cell carcinoma of the human urinary bladder. *Br. J. Cancer*, **63**, 967–970.

YAMAMOTO, T., IKAWA, S., AKIYAMA, T., SEMBA, K., NOMURA, N., MIYAMA, N., SAITO, T. & TOYOSHIMA, K. (1986). Similarity of protein encoded by human c-*erb*B-2 gene to epidermal growth factor receptor. *Nature*, **319**, 230–234.

ZINGG, E.J. & WALLACE, D.M.A. (1985). *Clinical Practice in Urology. Bladder Cancer*. Springer: Berlin.